# P-66. Patient Characteristics Associated with Risk for Hospital Onset Bacteremia

**DOI:** 10.1093/ofid/ofaf695.295

**Published:** 2026-01-11

**Authors:** Shruti K Gohil, Jennifer Yim, Thomas T Tjoa, Amarah Mauricio, Keith M Madey, Kathleen A Quan, Jennifer Cox, Susan Huang

**Affiliations:** University of California, Irvine, Irvine, CA; Epidemiology & Infection Prevention, UC Irvine Health, Orange, CA, Orange, California; University of California, Irvine School of Medicine, Division of Infectious Diseases, Irvine, California; Division of Infectious Diseases, University of California, Irvine School of Medicine, Irvine, California; University of California, Irvine, Irvine, CA; University of California, Irvine, Irvine, CA; University of California Irvine, School of Medicine, Irvine, CA, Division of Infectious Diseases, Irvine, California; University of California, Irvine School of Medicine, Irvine, California

## Abstract

**Background:**

Hospital onset bacteremia (HOB) is being considered as a patient safety metric during hospitalization. We evaluated patient characteristics associated with HOB and assessed what proportion of these events are captured by current infection prevention surveillance and performance improvement metrics.

**Figure:**
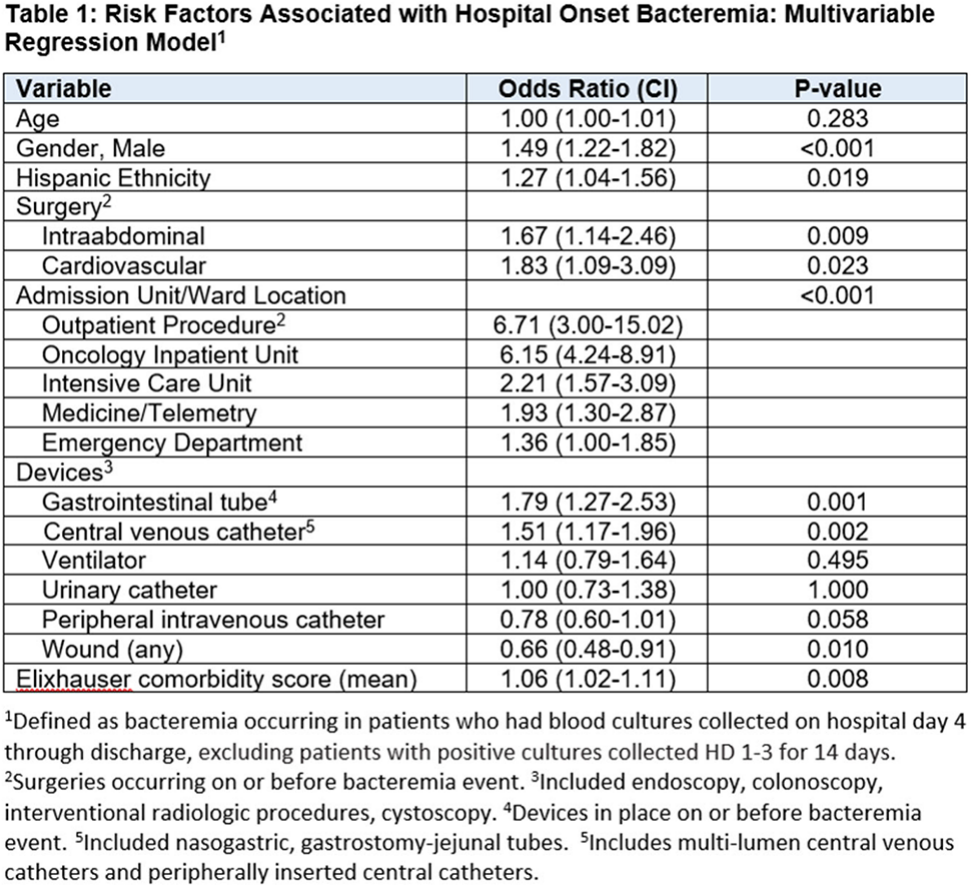

**Methods:**

This retrospective cohort study evaluated all patients admitted to a large academic medical center 1/1/2022-3/31/24 with length of stay (LOS) >3 days, assessing patients with positive blood cultures collected on hospital day (HD) 4 through discharge. We collected patient demographics, unit location on admission day, and comorbidities. The following were included if occurring on or before bacteremia date: presence of devices [e.g., venous/urinary catheters, ventilators, gastrointestinal (GI) tubes], outpatient procedures (e.g., endoscopy, colonoscopy, cystoscopy), and inpatient surgery on/before. We assessed the proportion of HOB events meeting CDC NHSN criteria for central line associated bloodstream infection (CLABSI) or catheter associated urinary tract infection (CAUTI) within 3 days before/after bacteremia or surgical site infection (SSI). Generalized linear mixed effects model assessed risk factors associated with likelihood of HOB.

**Results:**

Among 35,966 patients with LOS >3 days, 2,301 (6%) had blood cultures sent. Among patients cultured, 509 (22%) were positive and 427 (84%) had HOB, with mean (SD) age 57.3 (19.4), 269 (63%) were male, 187 (44%) were Hispanic, and 341 (80%) were Medicare/Medicaid insured. Among HOB patients, 103 (24%) met CLABSI, 22 (5%) met SSI, and 4 (0.9%) met CAUTI criteria. As shown in Table 1, HOB risk was 6.7-fold higher in patients who had an outpatient procedure, 6.15-fold higher if in oncology unit, and 2.2-fold higher if in the ICU. Risk was higher in those with gastrointestinal tube (1.79-fold) and central venous catheter (1.51-fold).

**Conclusion:**

Most HOB events are not captured by current healthcare-associated infection surveillance criteria. Potential performance improvement activities to reduce HOBs should target patients hospitalized after outpatient procedures, admitted to an oncology or ICU unit, or who have GI tubes or central lines.

**Disclosures:**

Susan Huang, MD, MPH, Xttrium: Conducting studies in which participating nursing homes and hospitalized patients receive contributed antiseptic products|Xttrium Laboratories: Conducting studies in which participating nursing homes and hospitalized patients receive contributed antiseptic product

